# Targeting Mitochondrial Metabolism to Reverse Radioresistance: An Alternative to Glucose Metabolism

**DOI:** 10.3390/antiox11112202

**Published:** 2022-11-07

**Authors:** Chenbin Bian, Zhuangzhuang Zheng, Jing Su, Huanhuan Wang, Sitong Chang, Ying Xin, Xin Jiang

**Affiliations:** 1 Jilin Provincial Key Laboratory of Radiation Oncology & Therapy, The First Hospital of Jilin University, Changchun 130021, China; 2 Department of Radiation Oncology, The First Hospital of Jilin University, Changchun 130021, China; 3 NHC Key Laboratory of Radiobiology, School of Public Health, Jilin University, Changchun 130021, China; 4 Key Laboratory of Pathobiology, Ministry of Education, Jilin University, Changchun 130021, China

**Keywords:** radioresistance, reactive oxygen species, oxidative phosphorylation, oncometabolites, apoptosis

## Abstract

Radiotherapy failure and poor tumor prognosis are primarily attributed to radioresistance. Improving the curative effect of radiotherapy and delaying cancer progression have become difficult problems for clinicians. Glucose metabolism has long been regarded as the main metabolic process by which tumor cells meet their bioenergetic and anabolic needs, with the complex interactions between the mitochondria and tumors being ignored. This misconception was not dispelled until the early 2000s; however, the cellular molecules and signaling pathways involved in radioresistance remain incompletely defined. In addition to being a key metabolic site that regulates tumorigenesis, mitochondria can influence the radiation effects of malignancies by controlling redox reactions, participating in oxidative phosphorylation, producing oncometabolites, and triggering apoptosis. Therefore, the mitochondria are promising targets for the development of novel anticancer drugs. In this review, we summarize the internal relationship and related mechanisms between mitochondrial metabolism and cancer radioresistance, thus exploring the possibility of targeting mitochondrial signaling pathways to reverse radiation insensitivity. We suggest that attention should be paid to the potential value of mitochondria in prolonging the survival of cancer patients.

## 1. Introduction

Cancer is a serious problem that threatens human life, and the number of cancer-related deaths and incidences are increasing annually. According to the 2020 World Cancer Report, 4.57 million new cancer cases and 3 million cancer-related deaths have occurred in China, ranking it first in the world for cases and deaths [1]. As a traditional cancer treatment, radiotherapy causes nuclear DNA damage directly via ionizing radiation (IR) or indirectly via the production of reactive oxygen species (ROS), pushing cancer cells with high levels of DNA damage over the threshold for cell death [2]. As some tumors, such as malignant lymphoma, testicular seminoma, and nephroblastoma, are highly sensitive to IR, an explosion of interest in the role of radiotherapy in eradicating tumor cells has been observed in recent decades [3,4,5]. Mitochondria exist in most cells and are the main sites of cellular aerobic respiration, adapting to rapid tumor growth demands by regulating the process of energy production [6]. It is worth noting that mitochondria are the most important targets of IR damage aside from the nucleus [7]. Radiation-induced mitochondrial DNA mutations and electron transport chain (ETC) disruption activate oxidative stress and eventually trigger the mitochondrial apoptosis pathway, which seriously affects the survival of tumor cells [8]. However, tumor cell resistance to IR remains an important obstacle that hinders the clinical application of radiotherapy, potentially leading to poor prognosis, tumor recurrence, and metastasis [9,10]. In addition, radioresistance increases the incidence of radiation-induced damage to normal tissue cells surrounding the tumor and the disruption of homeostasis, mainly manifesting as radiation pneumonitis, intestinal dysbiosis, hemorrhage, and cardiac-related complications [11,12]. Fractionated treatment regimens have been established for radiotherapy. As fractionation is the process of dividing a radiation dose into multiple fractions, fractionated radiotherapy ensures as much tumor cell death as possible while reducing normal tissue complications. However, IR has been shown to activate epithelial–mesenchymal transition transcription factors such as Snail, Slug, Twist, ZEB1/2, hypoxia-inducible factor 1 (HIF1), and signal transducer and transcriptional activator 3 (STAT3); interfere with glucose and mitochondrial metabolism; promote metastatic potential; and increase the likelihood of radioresistance [13,14,15].

Metabolic disorders have long been recognized as carcinogenic factors [16]. Metabolic reprogramming, the alteration in metabolic pathways by which cancer cells can proliferate rapidly, survive under conditions of nutrient depletion and hypoxia, and evade the immune system, is considered a hallmark of cancer [17].

Glucose is the primary energy source that drives the rapid proliferation of cancer cells, and cancer starvation therapy based on glucose deprivation to induce oxidative stress has become an effective method for inhibiting tumor growth and survival [18]. 2-Deoxy-D-glucose (2DG), a glucose analog, targets glucose metabolism to deplete energy in cancer cells [19]. For most cancer cells, 2DG treatment alone does not significantly induce cell death, but renders cells more vulnerable to the oxidative stress induced by radio- or chemotherapy [20]. For example, 2DG combined with cisplatin or radiation enhances the cytotoxicity of head and neck squamous cell carcinoma through metabolic oxidative stress [21]. Furthermore, inhibition of glycolysis (2DG) and intracellular redox metabolism (glutathione/thioredoxin) improves the radiation response of radioresistant cervical cancers [22]. Unexpectedly, glucose deprivation promotes the death of malignant cells and induces colorectal cancer migration, invasion, and epithelial–mesenchymal transition (EMT). Knockdown of thioredoxin-1 can decrease G6PD protein expression and activity thereby reducing NADPH production, increasing ROS levels, enhancing glucose-starvation-induced cell death, and reversing aggressive or metastatic potential during cancer progression [23]. Rapidly proliferating cells tend to have high G6PD activity, while the pentose phosphate pathway (PPP) is the main pathway for glucose catabolism, and its reductant NADPH can be used to detoxify intracellular ROS, thus acting as an antioxidant defense [24]. During oxidative stress, cancer cells selectively shut down the glycolytic pathway, thereby increasing the glucose flux through PPP to meet the need for NADPH synthesis [25]. Snail, a key transcriptional repressor of EMT, regulates the glucose flux between glycolysis and PPP by inhibiting the platelet isoform of phosphofructokinase (PFKP) expression, which plays an important role in cancer cell survival [26]. Thus, interfering with the PPP to disrupt NADPH homeostasis not only enhances radiotherapy-induced immunogenic cell death but also overcomes cisplatin resistance [27,28].

**Figure 1:**
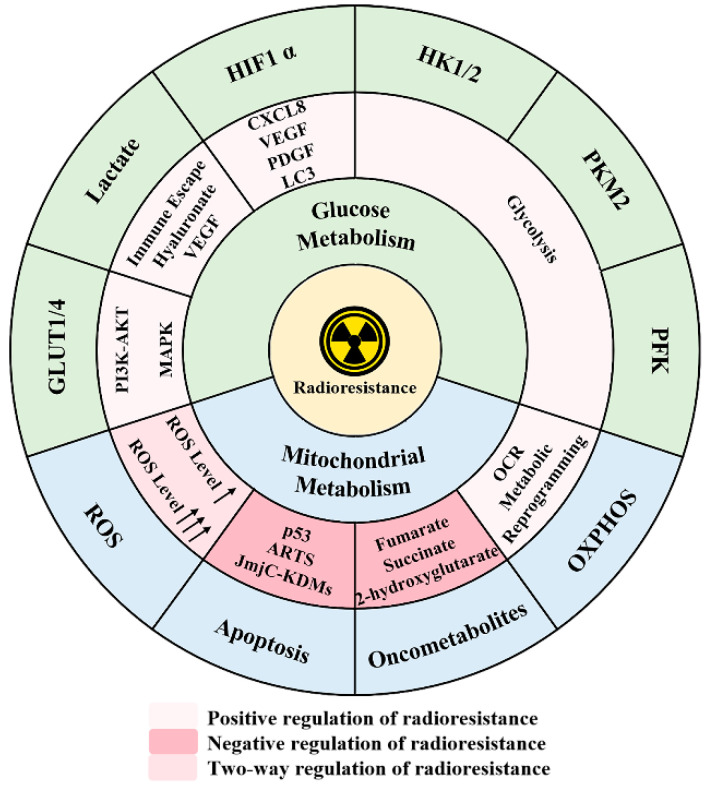
A schematic model illustrating the effects of two major metabolisms on radioresistance. Six targets in glucose metabolism have the most significant impact on radiation resistance, regulating their corresponding molecules or processes to intervene in the therapeutic effect. In general, small-molecule inhibitors can be used to help IR restore the expected efficacy, and there are several approved drugs currently available for clinical treatment. The effects of mitochondrial metabolism on radiation resistance can be summarized in four aspects, the details of which are presented below. Abbreviations: GLUT1/4—glucose transporter 1/4, PFK—phosphofructokinase, HK1/2—hexokinase 1/2, PKM2—pyruvate kinase M2, HIF1 α—hypoxia-inducible factor 1 α, OXPHOS—oxidative phosphorylation, ROS—reactive oxygen species.

Because of these classical conclusions, it was erroneously believed that malignant cells met their bioenergetic and anabolic needs primarily through glucose metabolism, and the role of mitochondrial metabolism in all steps of tumorigenesis was ignored [29]. However, the latest research has indicated that malignant transformation, tumor progression, and evasion of exogenous stress are influenced by mitochondria metabolism [30,31]. In addition, although the effect of radiation therapy is primarily dependent on glucose metabolism, there is growing awareness that changes in mitochondrial metabolism, such as mitochondrial function associated with antiradiation effects, also contribute to the development of radioresistance in head and neck squamous cell carcinomas and gliomas (Figure 1) [32,33]. Changes in mitochondrial size and shape or mutations in mitochondrial DNA interfere with the normal physiological function of mitochondria, thereby enhancing their adaptability to radiation [34]. Therefore, it is necessary to understand the molecular mechanisms underlying these changes caused by mitochondria to improve the efficacy of radiotherapy. Here, we briefly review the research progress on the relationship between mitochondrial metabolism and radioresistance from four aspects: regulating oxidative stress, participating in oxidative phosphorylation (OXPHOS), producing oncometabolites, and triggering apoptosis (Figure 1), with a focus on the possibility of targeting mitochondrial metabolism for cancer therapy.

## 2. ROS and Radioresistance

Reactive oxygen species are products of normal cellular metabolism and mainly include superoxide anions (O_2_^−^), hydrogen peroxide (H_2_O_2_), and hydroxyl radicals (^−^OH) [35]. Multiple lines of evidence indicate that there are three main sources of ROS in vivo, namely macrophages, the mitochondrial respiratory chain, and mitochondrial polyunsaturated membrane lipid peroxidation, a process during which ROS from mitochondrial polyunsaturated membranes pose the greatest threat to cells [36]. Under normal physiological conditions, cells tend to maintain redox homeostasis, that is, the balance between the production of free radicals and reactive metabolites (oxidants, ROS, or reactive nitrogen species) and their elimination through protective mechanisms (antioxidant systems) [37]. When the balance between ROS and antioxidants is disrupted, the body is in a state of oxidative stress, resulting in damage to important biomolecules and cells with potential effects on the entire organism [38]. It is worth noting that oxidative stress is generally present in tumor cells, and data show that the concentration of ROS is usually 10 times higher than that in normal cells, which may further lead to DNA mutation, genomic instability, and tumor cell proliferation [39].

Most ROS in mammalian cells are generated by the mitochondrial oxidative respiratory chain [40]. Furthermore, an inextricable relationship exists between ROS production and radioresistance. Mitochondrial H_2_O_2_ can trigger the accumulation of potential oncogenic DNA or activation of potential oncogenic signaling pathways, including the mitogen-activated protein kinase (MAPK) and epidermal growth factor receptor (EGFR) signaling pathways, thereby promoting cell proliferation and malignant transformation [41,42]. Experiments have demonstrated that activation of the MAPK and EGFR signaling pathways can increase the resistance of cervical and lung cancer cells to radiation, respectively, and knockout of thyroid hormone receptor interactor 4 (TRIP4) promotes the inactivation of MAPK signaling, which effectively improves the sensitivity of the former to radiation [43,44]. It has also been reported that activated O_2_^−^- and H_2_O_2_-mediated cell survival in non-small-cell lung cancer (NSCLC) occurs via the c-Met-PI3K-Akt and c-Met-Grb2/SOS-Ras-p38 pathways [45]. Interestingly, acidosis is a common characteristic of the tumor microenvironment. Under such acidic conditions, the specific mitogen reaction of cancer cells reduces extracellular acidification and increases O_2_^−^ production by switching from glycolysis to OXPHOS, which promotes tumor invasiveness and insensitivity to radiation therapy [31]. However, the mechanism appears to be different in endothelial cells, where mitochondrial ROS (mtROS) can stimulate the activity of NAD(P)H oxidases (NOXs), resulting in a positive feedback loop of ROS-induced ROS generation [46]. Recent studies have also shown that NOXs significantly contribute to H_2_O_2_ and O_2_^−^ production in gastrointestinal and pancreatic cancers [47,48]. Kim et al. reported that the novel PPARɣ ligand PPZ023 can lead to NOX4-derived mtROS generation to induce death of radioresistant NSCLC cells via exosomal endoplasmic reticulum stress [49].

**Figure 2:**
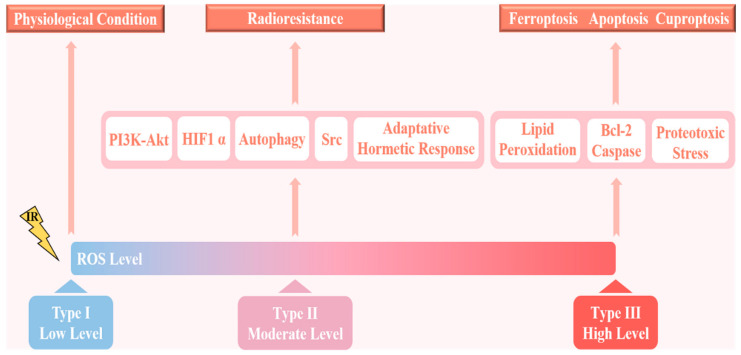
A schematic diagram of the different physiological effects of varying ROS concentrations. Under physiological conditions, ROS levels are maintained at a low state under the regulation of antioxidant systems, which ensures the survival of normal cells. Moderate levels of ROS tend to promote tumor initiation and progression, and to some extent interfere with the efficacy of radiotherapy. A continued rise in ROS levels to high levels will lead to cell death.

The reason for these diametrically opposite results may be the dual role of ROS, in which the difference in the level of ROS is dominant (Figure 2). In normal cells, ROS are produced at low concentrations and are effectively neutralized by the potent antioxidant systems of the cells. A moderate increase in ROS levels as in states of chronic oxidative stress induces random mutations in cells and promotes tumor cell proliferation, metastasis, and radioresistance. If ROS levels continue to increase beyond the antioxidant capacity of cells, this will cause apoptosis, ferroptosis, or cuproptosis, thereby significantly improving the efficacy of radiotherapy [38,50]. Sublethal levels of ROS stimulate tumor cell proliferation by inhibiting tumor suppressors such as redox-sensitive phosphatase and tensin homologues (PTEN), thereby promoting the PI3K-Akt signaling pathway or stabilizing HIF1 α, and are associated with chemotherapy resistance and prevention of tumor cell death [51]. In addition, a slight increase in superoxide can activate signal transduction pathways related to metastasis, including the mtROS-Src-SMAD-Pyk2 signaling pathway; in particular, Src can also promote radiation resistance in glioblastoma (GBM) [52,53]. Combining the novel Src inhibitor Si306 with radiotherapy represents a promising approach to increasing the therapeutic effect on GBM [54]. Importantly, moderately elevated ROS levels increase the resistance of cancer cells to radiotherapy by triggering an adaptive hormetic response and promoting autophagy activation [55,56]. Conversely, in the case of severe oxidative stress, ROS cause regulated cell death (RCD) or trigger apoptosis independently of DNA damage, thereby increasing the sensitivity to radiotherapy (Figure 2) [57]. For example, elesclomol (STA-4785) targets tumor ROS, which can further increase ROS levels in tumor cells, induce cytotoxicity in tumor cells, and selectively induce apoptosis in melanoma cells [58]. Unexpectedly, elesclomol did not show a significant radiosensitization effect on prostate cancer cells, indicating that there was no clear linear relationship between the specific ROS dose and radioresistance [59]. Of course, we should not ignore the fact that early and late ROS accumulation can lead to opposite carcinogenic effects. Radiation-induced early ROS signaling is responsible for the activation of Jak3-Erk-STAT3, which leads to a cell survival response, whereas late ROS production is different [60].

In cells, ROS production is counterbalanced by cellular antioxidant defense systems. Superoxide dismutases (SODs), the most potent antioxidant enzymes in mitochondria, can catalyze O_2_^−^ to H_2_O_2_ [61]. SOD-produced H_2_O_2_ can be subsequently reduced to H_2_O by catalases (CATs), glutathione peroxidases (GPXs), and peroxiredoxins (Prxs) [31]. To date, an increasing amount of evidence has suggested that the antioxidant stress system is responsible for radio- and chemoresistance [38]. Furthermore, ROS induced by chemoradiotherapy activate the Keap1-Nrf2 and PI3K-AKT pathways, which regulate several antioxidant enzymes in downstream signaling, ultimately triggering both radio- and chemoresistance [62,63]. The inhibitors of these two signaling pathways, trigonelline and delicaflavone, can significantly reverse radioresistance and enhance radiosensitivity, further demonstrating the detrimental effects of the antioxidant stress system on cancer therapy [64,65]. Studies have shown an important relationship between an increase in the survival rate of pancreatic cancer cells after γ-ray irradiation and enhancement of the activity of manganese superoxide dismutase (MnSOD), the main antioxidant enzyme in the body, which also indicates that MnSOD significantly increases the resistance of pancreatic cancer to radiotherapy [66]. CuZn-SOD overexpression confers radioresistance on human glioma cells by suppressing irradiation-induced late ROS accumulation (superoxide) [67]. GPX4 inhibition promotes lipid peroxidation and re-sensitizes radioresistant cancer cells to IR-induced ferroptosis, resulting in radiosensitization [68]. In addition, redox-active metal ions are involved in antioxidant reactions, such as O_2_^−^- and H_2_O_2_-mediated disruption of Fe metabolism, sensitizing NSCLC and GBM to pharmacological ascorbate [69]. However, recent studies have yielded conflicting results that antioxidant supplementation is detrimental to patients with adequate antioxidant status (lung, gastrointestinal tract, head and neck, and esophagus), whereas individuals with deficient antioxidant systems respond positively [47].

Based on the above studies, it can be concluded that ROS is a double-edged sword; it is one of the ways in which radiotherapy can eradicate tumor cells, yet even moderate intracellular concentrations may lead to radioresistance. Although the dual role of ROS is a major challenge in cancer therapy, it presents a promising strategy to differentiate normal cells from cancer cells using specific cellular signals to target tumor killing. An in-depth understanding of the dynamic balance between ROS and antioxidant levels and the role of ROS in different stages of the disease will help researchers to develop personalized therapies for different tumor types. Both disabling cellular antioxidants and adding specific ROS inducers provide new ideas for the precise treatment of tumors and the improvement in radiosensitivity.

## 3. OXPHOS and Radioresistance

Carbohydrates are the main source of cellular energy and are involved in the oxidative breakdown of glucose including glycolysis and OXPHOS [70]. Normally, cells favor the application of the mitochondrial OXPHOS, which is more efficient at producing ATP; however, the rate of glucose metabolism by aerobic glycolysis is 10–100 times faster than that of the complete oxidation of glucose in the mitochondria [71]. Therefore, Warburg initially believed that cancer cells could have an active glycolytic phenotype even in the presence of adequate oxygen supply and completely functioning mitochondria [72]. Warburg later proposed that this phenomenon was due to a developmental defect in the mitochondria of tumor cells that resulted in impaired aerobic respiration and reliance on glycolysis, hypothesizing that this event was the primary cause of cancer [73,74]. In contrast, Koppenol et al. offered a more plausible explanation, emphasizing the impairment of glycolytic regulation rather than mitochondrial respiration. There are clear indications that glycolysis is upregulated in most tumors without mitochondrial dysfunction. In these cancers, OXPHOS continues normally, even producing as much ATP as normal tissue at the same partial pressure of oxygen [75]. Notably, Weinhouse also strongly criticized the Warburg effect for his finding that well-differentiated Morris hepatomas do not produce lactic acid in aerobiosis [76]. At the same time, metabolic changes and adaptations occurring in tumors have been demonstrated to extend well beyond the Warburg effect and are seen as a secondary effect of tumorigenesis [77]. Subsequent studies have further revealed that certain types of tumors such as ovarian cancer and acute myeloid leukemia can also rely on mitochondria-specific OXPHOS to maintain biosynthesis and bioenergetics in addition to glycolysis [78]. Furthermore, in B16 melanoma the Warburg effect has been shown to be dispensable owing to the upregulation of mitochondrial metabolism [79].

**Figure 3:**
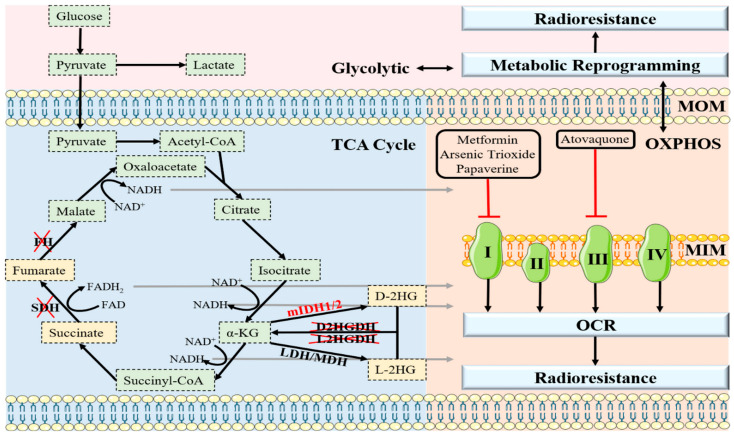
A schematic summary of glycolysis, TCA cycle, and OXPHOS in regulating radioresistance. (1) In the cytoplasm, pyruvate produced by glycolysis crosses the outer mitochondrial membrane and participates in the TCA cycle, and the subsequently produced NADH and FADH_2_ are oxidized by a stepwise, continuous enzymatic reaction on the ETC located in the inner mitochondrial membrane, thereby releasing energy for the body to utilize. (2) Inactivation of FH, SDH, D2HGDH, and L2HGDH, mutation of IDH1/2, or promiscuous activity of MDH/LDH can induce accumulation of oncometabolites. On the one hand, oncometabolites promote tumorigenesis; on the other hand, they also amplify the benefits of radiotherapy. (3) Enhancing OCR or favoring the reprogramming of tumor cells’ metabolic pathways induces radioresistance. Abbreviations: TCA cycle—tricarboxylic acid cycle, ETC—electron transport chain, OCR—oxygen consumption rate, FH —fumarate hydratase, SDH—succinate dehydrogenase, D2HGDH—D-2-hydroxyglutarate dehydrogenase, L2HGDH—L-2-hydroxyglutarate dehydrogenase, IDH1/2—isocitrate dehydrogenase-1/-2, LDH/MDH—lactate dehydrogenase/malate dehydrogenase, α-KG—α-ketoglutarate, D-2HG—D-2-hydroxyglutarate, L-2HG—L-2-hydroxyglutarate.

Based on the above findings, we can conclude that metabolic reprogramming endows cancer with the ability to utilize multiple metabolic modalities to rapidly progress in vivo [80]. Some studies indicate that metabolic plasticity allows cells to efficiently produce energy through multiple metabolic pathways, thereby conferring on cancer cells a high degree of adaptability to a wide range of stresses and harsh tumor microenvironments [31]. In other words, tumor tissues are less sensitive to conventional chemoradiotherapy. In cancer cells that rely on glycolytic metabolism, OXPHOS can promote resistance to therapy through both the cancer-cell-intrinsic and -extrinsic pathways. In contrast, tumor cells that primarily utilize OXPHOS for energy production can become resistant to ETC inhibitors because they gain partial glycolytic metabolism (Figure 3) [81]. For example, recent studies have shown that acquired radioresistance is associated with a switch from glycolytic to oxidative metabolism in laryngeal squamous cell carcinoma cancer cells [32]. Similarly, a switch from glycolysis to OXPHOS was observed in glioma cells that developed acquired resistance to PI3K inhibitors [82]. In addition, glycolytic-dependent BRAF-mutant melanoma cells are more sensitive to the BRAF inhibitor vemurafenib, while resistant cells display upregulation of the mitochondrial biogenesis co-activator PGC1α through the melanocyte master regulator microphthalmia-associated transcription factor (MITF), leading to resistance to the original treatment and sensitivity to OXPHOS inhibitors [83]. Interestingly, glucose deprivation significantly promotes mitochondrial elongation, thereby inducing a metabolic shift from glycolysis to OXPHOS during energy stress in tumor cells, which is critical for hepatocellular carcinoma (HCC) survival [84]. Additionally, Dynamin-related protein 1 (DRP1) is necessary for mitochondrial elongation in HCC cells. Elongated mitochondria amplify OXPHOS through facilitating cristae formation and assembly of respiratory complexes and in turn, exerting a feedback inhibitory effect on glycolysis through NAD-dependent SIRT1 activation [84]. Consistent with this, nutrient-deprivation-related OXPHOS/glycolysis interconversion has also been observed in glioma cell lines, although the role of mitochondrial dynamics has not been investigated [85]. The only exception is that dichloroacetate, which activates OXPHOS by reversing aerobic glycolysis, improves the radiosensitivity of high-grade gliomas [86]. In conclusion, the vast majority of malignant cells can switch freely between the two metabolic modes, simply inhibiting glycolysis or OXPHOS as a reasonable therapeutic candidate. The combination of a glycolysis inhibitor (2-DG) with an OXPHOS inhibitor (metformin) significantly enhances the radiosensitization of neuroblastoma and glioma cells, suggesting that dual metabolic targeting may be a good strategy to control tumor progression and eliminate radioresistance [87]. Unfortunately, the cytotoxic effect of this combination on normal tissue remains the biggest obstacle to its clinical application.

Another mitochondrial condition of interest is hypoxia, which poses a problem for radiation therapists because the scarcity of oxygen induces radioresistance [88]. Efforts to increase oxygen delivery to tumors have not shown positive clinical effects because of poor tumor vascularization [89]. This implies that attempts to target tumor hypoxia should focus on normalizing oxygen levels in remote tumor regions by reducing the oxygen consumption rate (OCR). Therefore, an attractive strategy is to achieve this by inhibiting mitochondrial OXPHOS as it reduces the OCR, increases oxygenation, and thus improves the radiation response [90,91]. Several clinical trials are underway to repurpose FDA-approved drugs to curb mitochondrial function and reverse radioresistance. The antidiabetic drugs metformin and phenformin have been shown to increase the partial pressure of oxygen (pO_2_) in local tumors by inhibiting mitochondrial complex I, thereby significantly improving the effect of radiotherapy on colorectal cancer cells [92]. Another complex I inhibitory molecule, arsenic trioxide (As_2_O_3_), has shown strong superiority in the treatment of acute promyelocytic leukemia. However, in recent years, more attention has been paid to the potential of As_2_O_3_ to overcome radioresistance in solid tumors [93]. Both OXPHOS levels and the OCR are impaired by As_2_O_3_ to varying degrees in liver and lung cancer cells, with enhanced radiosensitivity [94]. Papaverine, a smooth muscle relaxant used as a vasospasm and erectile dysfunction agent, not only leads to reduced hypoxia and an increased response to radiotherapy in NSCLC and breast cancer by blocking complex I, but also has significantly fewer side effects than other OXPHOS inhibitors [95]. Atovaquone was originally used to treat and prevent parasitic infections; however, in hypopharyngeal, colorectal, and lung cancer cell lines, it significantly increased oxygenation and sensitized tumors to radiotherapy by inhibiting electron transport complex III (Table 1) [96].

In addition to the above-mentioned drugs that have been investigated in clinical trials as hypoxia regulators, there has also been an explosion of interest in small molecules that have the potential to overcome radioresistance. For instance, since annonacin is a natural lipophilic inhibitor of complex I, in addition to its known ability to promote selective cancer cell death through NKA- and SERCA-dependent pathways, it is reasonable to speculate that annonacin may also act as a radiosensitizer through its potential ability to target OXPHOS [103,105]. In addition, experiments have shown that the anthelmintic pyrvinium pamoate inhibits the proliferation of myeloma, erythroleukemia, and pancreatic cancer cells by targeting mitochondrial respiratory complex I [98,106]. Considering this, IR combined with pyrvinium pamoate is a promising future direction for addressing the unsatisfactory effects of radiotherapy on radioresistant pancreatic cancer cells (Table 1). However, further clinical research is needed to clarify various issues that may be overlooked by new treatments, such as balancing the relationship between therapeutic effects and toxic side effects.

Given that OXPHOS involves two distinct modalities that interfere with the radiation response along with active metabolic reprogramming activity or persistent local hypoxia in some tumors, targeting mitochondrial respiration to overcome radioresistance has attracted attention. Indeed, as the therapeutic index is the decisive factor for the utility of any therapy, those targeting OXPHOS are often limited by side effects rather than a lack of efficacy; therefore, there is an urgent need to find novel and more specific radiosensitizers.

## 4. Oncometabolites and Radioresistance

Oncometabolites, defined as metabolites that accumulate abnormally from distorted metabolic pathways, play pivotal roles in tumor transformation, cancer progression, invasiveness, and therapy resistance [107]. Mutations in the genes encoding isocitrate dehydrogenase 1/2 (IDH1/2) or promiscuous activity of lactate dehydrogenase/malate dehydrogenase (LDH/MDH) leads to the synthesis of D-2-hydroxyglutarate (D-2HG) and L-2-hydroxyglutarate (L-2HG), respectively [108]. Furthermore, loss of function of the tricarboxylic acid cycle enzymes succinate dehydrogenase (SDH) and fumarate hydratase (FH) results in the accumulation of succinate and fumarate (Figure 3) [109]. Oncometabolites act as structural mimics of α-ketoglutarate (α-KG) and thus competitively interfere with α-KG-dependent dioxygenases, which are involved in regulating the demethylation status of histones, RNA, and DNA, and targeting HIF-α degradation [110,111]. For example, oncometabolites lead to extensive hypermethylation of histone 3 lysine 9 (H3K9me3) by inhibiting histone lysine demethylase (KDM), which hinders the recruitment of DNA repair factors, leading to genomic instability that promotes tumor growth [112]. Thus, fumarate, succinate, D-2HG, and L-2HG have been characterized as bona fide tumor metabolites and have become pathognomonic hallmarks of a growing number of cancers (Figure 3), including neuroendocrine tumors, gliomas, leukemia, renal cell carcinomas, and head and neck squamous cell carcinomas [113,114,115,116]. In recent years, there has been great interest in the possible role of oncometabolites in cancer cell resistance to radiation, and numerous clinical trials have been conducted.

Furthermore, IDH1/2 mutations have been predicted in clinical trials and retrospective analyses to improve the response to radiotherapy in low-grade gliomas, showing significantly prolonged progression-free survival and overall survival [117]. However, mutated IDH1, when co-expressed with inactivating TP53 and alpha thalassemia/mental retardation syndrome X-linked gene mutations in gliomas, induces genome stability and enhances the DNA damage response, triggering resistance to IR [118]. Thus, pharmacological inhibition of the DNA repair pathway is necessary if radiotherapy demonstrates superior therapeutic advantages in IDH1/2-mutated glioma cells. Studies have shown that FH expression in gastric cancer cells is significantly higher than that in nearby normal cells and is negatively correlated with patient prognosis. In addition, cisplatin is the first-line treatment for gastric cancer, and FH can significantly inhibit the cytotoxicity of cisplatin. Recent experiments have concluded that miconazole nitrate enhances the effects of cisplatin in vitro and in vivo by inhibiting FH activity [119]. Given that activated FH restrains sensitivity to traditional chemotherapy drugs, it could have the same adverse effects on radiation therapy. Patients with hereditary leiomyomatosis, renal cell cancer (HLRCC), and a substantial accumulation of fumarate are susceptible to kidney cancer with type 2 papillary morphology, which is refractory to current radiotherapy [120,121]. Interestingly, fumarate can covalently modify GPX4 and inhibit its activity, thereby activating ferroptosis-selective HLRCC cell death [122]. Furthermore, SDH5 is required for the activity of the SDH complex, and its rapid depletion inhibits p53 degradation through the ubiquitin/proteasome pathway, thereby promoting apoptosis and enhancing NSCLC radiosensitivity [123].

Taken together, it is not difficult to see this as an interesting phenomenon, and while oncometabolites are beneficial for cancer progression, they also appear to significantly reverse the resistance of tumor tissue to IR, likely because tumors that accumulate high levels of oncometabolites are more vulnerable to therapies that cause DNA damage [124]. Moreover, it has been demonstrated that KDM induces transforming growth factor (TGF)-β2 transcriptional activation by downregulating the enrichment of H3K9me3 at its promoter region. Activated TGF-β2 further enhances Smad/ATM/Chk2 signaling, which confers radioresistance in lung cancer [125]. Therefore, oncometabolites may be suitable signals indicative of radiosensitivity, providing new insights into possible methods for predicting radiotherapy responses in patients who cannot tolerate biopsy.

## 5. Apoptosis and Radioresistance

Apoptosis is a tightly controlled mode of programmed cell death that plays an essential role in development, tissue homeostasis, and defense against unwanted, redundant, and potentially dangerous cells, particularly in the regulation of tumorigenesis [126,127]. The mitochondrial apoptotic pathway initiated by caspases and regulated by members of the Bcl-2 family of proteins or inhibitors of apoptotic proteins may have particularly relevant roles in radiation signal transduction. In differential expression analysis of related genes in cervical cancer cell lines, 33 genes have shown changes in expression after radiation induction, which may have potential effects on the apoptosis of cervical cancer cells after radiotherapy [128]. These findings suggest that IR can lead to significant changes in the expression of apoptosis-related genes, thereby inducing radioresistance. Some genetic dysregulation, as commonly observed in apoptotic signaling pathways in aggressive cancer cells, greatly limits the efficacy of anticancer treatments such as radiotherapy, which relies on these pathways to eradicate tumors [129]. Enhancing apoptosis to improve the therapeutic effect in cancer can be accomplished in two ways: by upregulating pro-apoptotic genes or by interfering with anti-apoptotic protein function [130].

We have summarized the apoptotic molecules associated with radiation resistance, and insights into the mechanisms involved can guide subsequent therapeutic approaches as follows. (1) NF-κB is increasingly recognized as a key player in many steps from cancer initiation to progression, with some degree of activation in various tumors, such as gastric, colorectal, lung, nasopharyngeal, and prostate carcinomas [131,132,133,134]. The activity of NF-κB is often enhanced by radiation and plays a central role in the resistance of cancer cells to radiation through the activation of the pro-survival proteins Bcl-2 and Bcl-XL in downstream signaling pathways [135]. Curcumin, one of the most important inhibitors of NF-κB, significantly delays tumor regeneration in irradiated mice [136]. (2) p53, a tumor suppressor gene, also has a diametrically opposite significance in the development of radioresistance. Downregulation of p53-induced death-domain-containing protein expression and inhibition of ataxia–telangiectasia-mutated protein (ATM) directly silence NF-κB, which inhibits DNA damage repair and ultimately increases the radiosensitivity of tumor cells [129]. Conversely, radiation-induced DNA damage can also activate the downstream effector kinase Chk2 of ATM, which contributes to further activation of p53 and pro-apoptotic proteins PUMA and BAX to induce apoptosis [137]. A study showed that the loss of components in the ATM/Chk2/p53 pathway was associated with radioresistance in a glioma mouse model [138]. Radiotherapy, the standard treatment for patients with nasopharyngeal carcinoma (NPC), induces DNA methyltransferase 3B, which greatly contributes to radioresistance in NPC by methylating p53 and p21 [139]. (3) Apoptosis-related proteins in the TGF-β signaling pathway (ARTS) are alternative spliceosomes of the Sept4 gene located in the outer mitochondrial membrane [140]. As the only dual pro-apoptotic protein in vivo, ARTS directly bind to and restrain XIAP and Bcl-2 and assist p53 in inhibiting Bcl-XL [141]. Therefore, targeting the ARTS-mediated degradation of anti-apoptotic proteins may represent an effective way of sensitizing tumor cells to radiotherapy. (4) Most patients with breast cancer treated with radiotherapy are completely cured, but in partial IR-induced triple-negative breast cancer, activated STAT3 and Bcl-2 and reduced ROS promote cell proliferation, reduce apoptosis, increase angiogenesis, and increase immune evasion, thus severely compromising the effectiveness of radiotherapy [142]. Niclosamide, a small-molecule STAT3 inhibitor, leads to a significant decrease in the protein levels of downstream anti-apoptotic target genes (such as Bcl-XL and survivin) by inhibiting Tyr-705 phosphorylation and nuclear translocation of STAT3, thereby improving the survival of patients with radioresistant breast cancer [142,143]. (5) Recent studies have shown that amplification of the cancer-associated gene YWHAZ is an indicator of poor prognosis in patients with urothelial carcinoma of the bladder (UCUB) [144]. Upregulation of YWHAZ resulted in insufficient expression of pro-apoptotic proteins (BAK and BAX) and several caspases (CASP 3, 7, and 10) involved in mitochondrial apoptotic cascade reactions, with an emphasis on radiation insensitivity [145]. Notably, gene knockdown using a specific shRNA triggered a significant increase in cell death after radiation therapy, providing a new therapeutic target for YWHAZ-overexpressing UCUB [145].

**Figure 4:**
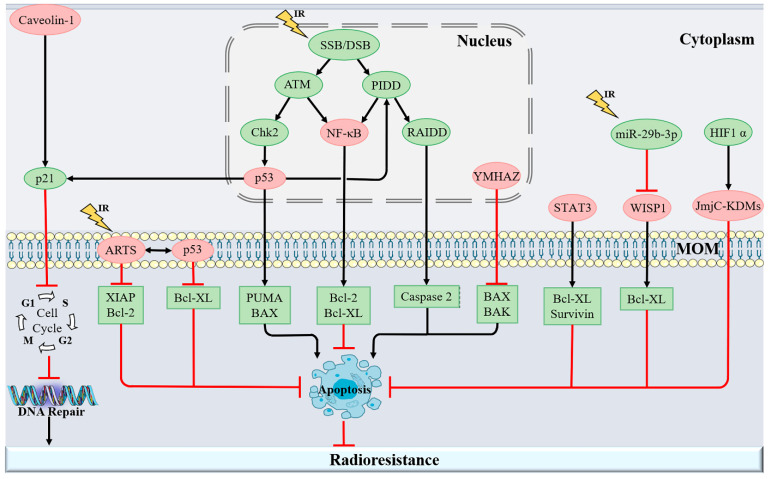
A schematic representation of the signaling mechanism of apoptosis-related molecules. IR induces DNA damage, leading to abnormal expression of mitochondria-related proteins, or directly regulates apoptosis-related genes, thereby promoting DNA damage repair and inhibiting mitochondrial apoptosis, ultimately causing the occurrence of radioresistance. Abbreviations: SSB/DSB—single-strand breaks/double-strand breaks, ATM—ataxia–telangiectasia-mutated protein, PIDD—p53-induced death-domain-containing protein, Chk2—checkpoint kinase 2, NF-κB—nuclear factor-κB, RAIDD—receptor-interacting protein (RIP)-associated ICH-1/CED-3-homologous protein with a death domain, ARTS—apoptosis-related proteins in the TGF-β signaling pathway, STAT3—signal transducer and transcriptional activator 3, WISP1—Wnt1-inducible-signaling protein 1, JmjC-KDMs—Jumonji C domain histone lysine demethylases.

In addition, Jumonji C domain histone lysine demethylases (JmjC-KDMs), Wnt1-inducible-signaling protein 1 (WISP1), and Caveolin-1 can also interfere with apoptosis and further induce radioresistance [146,147,148] (Figure 4). Therefore, elucidating the precise mechanism underlying the interaction between mitochondrial apoptosis and radioresistance would benefit the development of novel radiosensitizers. Although drugs developed based on this principle still require more clinical experiments to verify their indications and safety, they undoubtedly provide a promising starting point for the treatment of tumors with high target gene expression levels.

## 6. Conclusions and Perspectives

Acquired radioresistance is the main clinical obstacle for patients with tumors receiving radiotherapy and is affected by several factors. Since the Warburg effect was proposed, glucose metabolism has received unprecedented attention. However, a considerable number of studies point to the development of radioresistance closely related to mitochondrial metabolism, not only because mitochondria predominate in the tolerance of malignant cells to radiation-induced RCD, but also because it underlies metabolic reprogramming. Maintaining the normal physiological function of the mitochondria is an important factor that improves the effect of radiotherapy. To date, many small-molecule inhibitors have been developed against ROS and oncometabolites or to regulate OXPHOS and apoptosis, which can target specific receptors and enhance the radiation response of tumor tissue. However, owing to the lack of high specificity, the indiscriminate attack of radiosensitizers on non-tumor cells can have unwanted effects, which also hinders their generalization. Due to different tumor types and specific metabolic processes or molecules, we need to individualize the treatment of tumors, so the development of more effective and specific sensitizers has become an irreplaceable solution. Despite these challenges, with a deeper understanding of the mechanism of radioresistance, targeting mitochondrial metabolism to reverse radiation insensitivity may be a safe and efficient radiosensitizing method in the future and thus deserves more attention.

## Figures and Tables

**Table 1:** List of OXPHOS inhibitors under clinical trials or potential.

OXPHOS Inhibitor	Identifier	Phase	Cancer Type	ECT Target	Ref.
Metformin	NCT04275713, NCT04414540, NCT04945148, NCT04387630	II, II, II, II	Cervical cancer, head and neck squamous cell carcinoma, glioblastoma (IDH-wildtype), breast cancer	Complex I	[92]
Phenformin	NCT03026517	I	Melanoma	Complex I	[92]
Arsenic Trioxide	NCT02066870, NCT03503864	I, II	Non-small-cell lung cancer, neuroblastoma	Complex I	[93]
Papaverine	NCT05136846, NCT03824327	I, I	Locally advanced or unresectable non-small-cell lung cancer	Complex I	[95]
Atovaquone	NCT04648033, NCT02628080	I, I	Locally advanced non-small-cell lung cancer, non-small-cell lung cancer	Complex III	[96]
Proguanil	N.A.	N.A.	Acts synergistically with atovaquone	Complex I	[97]
Pyrvinium Pamoate	NCT05055323	I	Resectable pancreatic ductal adenocarcinoma	Complex I	[98]
Vitamin E	NCT01871454	II	Non-small-cell lung cancer	Complex II	[99]
ONC201	NCT04055649	II	Platinum-resistant epithelial ovarian, fallopian tube, or primary peritoneal cancer, diffuse midline gliomas	Complex I, II	[100]
Mitoxantrone	NCT04927481, NCT03839446, NCT03258320, NCT04718402	II, II, I, I	Breast cancer, acute myeloid leukemia, prostate cancer patients, advanced gastric carcinoma	Complex V	[101]
Ivermectin	N.A.	N.A.	Induces the death of renal cancer cells, chronic myeloid leukemia cells, and glioblastoma cells *	Complex I	[102]
Anonacin	N.A.	N.A.	Delays the growth of pancreatic cancer cells *	Complex I	[103]
Trifluoperazine	N.A.	N.A.	Induces pancreatic ductal adenocarcinoma cell death in combination with bortezomib *	Mitochondrial Stress	[104]

* indicates that the drug has not been validated by clinical trials but has been confirmed in in vitro cell experiments and in vivo xenograft models.
